# Working Conditions and Long-Term Sickness Absence Due to Mental Disorders: A Prospective Record Linkage Cohort Study Among 19- to 39-Year-Old Female Municipal Employees

**DOI:** 10.1097/JOM.0000000000002421

**Published:** 2021-10-28

**Authors:** Noora Heinonen, Tea Lallukka, Jouni Lahti, Olli Pietiläinen, Hilla Nordquist, Minna Mänty, Anu Katainen, Anne Kouvonen

**Affiliations:** Faculty of Social Sciences, University of Helsinki, Finland (Heinonen, Dr Nordquist, Dr Katainen, and Dr Kouvonen); Department of Public Health, Faculty of Medicine, University of Helsinki, Finland (Dr Olli, Dr Nordquist, and Dr Mänty); South-Eastern Finland University of Applied Sciences, Kotka, Finland (Dr Nordquist); Department of Public Health, Faculty of Medicine, University of Helsinki, Finland; and Unit of strategy and research, City of Vantaa, Vantaa, Finland (Dr Mänty); Centre for Public Health, Queen's University Belfast, UK (Dr Kouvonen).

**Keywords:** mental disorders, physical working conditions, psychosocial working conditions, sickness absence, young employees

## Abstract

**Objective:**

We examined associations between working conditions and long-term sickness absence due to mental disorders (LTSA-MD) among younger female public sector employees from different employment sectors.

**Methods:**

Survey data collected in 2017 (*n* = 3048) among 19- to 39-year-old female employees of the City of Helsinki, Finland, were used to examine job demands, job control, physical workload, computer work, and covariates. Register data on LTSA-MD were used over 1-year follow-up. Negative binomial regression models were applied.

**Results:**

Adverse psychosocial and physical working conditions were associated with higher LTSA-MD during the follow-up. Health and social care workers had the highest number of days of LTSA-MD.

**Conclusion:**

Working conditions are important factors when aiming to prevent LTSA-MD among younger employees, in the health and social care sector in particular.

**Learning Objectives**

- Discuss previous knowledge on mental disorders as a cause of work disability in younger adults, including differences by sex and employment sector.
- Summarize the new findings on long-term sickness absence due to mental disorders (LTSA-MD) among younger female employees in Finland, including associations with psychosocial and physical working conditions
- Discuss the implications for prevention of LTSA-MD among younger employees, particularly in higher-risk occupational sectors.

Mental disorders have become the leading cause for work disability across Western countries with most of the disease burden related to depression and anxiety.^1–3^ Of particular concern are younger adults, among whom work disability due to mental disorders, as expressed by sickness absence and disability pensions, have been rising alarmingly over the past two decades.^3–6^ In Finland, for example, the number of sickness allowances granted for mental disorders increased in the youngest employee groups (16 to 34 year olds) by 57% for women and by 48% for men just in three years, between 2016 and 2019.^7^ Younger employees have been underrepresented in work ability research^8^ and have been examined mostly as part of the general working-age population.^9–11^ However, predictors of work disability can vary by age^9^ and there appers to be differences in the patterns of sickness absence among younger and older employees.^12^

Mental disorders are multi-factorial by etiology, and several socioeconomic, demographic, biological and environmental risk factors have been associated with a higher risk of long-term sickness absence due to mental disorders (LTSA-MD). These include female gender,^9,10,13–18^ marital status other than cohabiting or married,^10^ lower socioeconomic position (SEP),^10,14,19–22^ comorbidity,^23^ poor general health,^14^ and adverse health behaviors.^14,17,23^ The evidence suggests that the links between mental ill-health, socioeconomic status, and the exposure to unfavourable working conditions can go back to early life stages. Poor mental health in childhood and adolescence can lead to worse educational attainment and thereby influence the selection into jobs with more adverse working conditions.^24^ In addition, being employed in the public sector compared to the private sector has been associated with a higher risk of LTSA-MD.^10^ Previous studies have also indicated a twofold risk of LTSA-MD for employees in the health and social care sector and other human service work compared to other occupations.^25–28^

The contribution of working conditions to the risk of SA-MD is also widely recognized in the literature especially from the perspective of psychosocial working conditions,^29^ which are most often assessed by the control-demand-support model.^30^ Longitudinal studies indicate that high job demands,^17^ low job control, and their combination ‘high job strain,^17,18^ and a combination of high strain and low support^23^ are associated with an increased risk of LTSA among employees. However, there are some inconsistencies in the literature as not all studies have supported this link: No association was found in either between low control nor high job demands and LTSA-MD among working-age Swedish health care workers,^31^ or among Belgian private and public sector employees.^32^ In Brazilian workforce, no associations were observed between high job strain or low support and LTSA-MD^33^ Furthermore, a recent Finnish longitudinal study found no associations of high job demands or low job control with work disability due to mental disorders (measured by sickness absence and disability pension) in young, middle aged or older public sector employees in Finland.^9^

Only few studies have examined the associations between physical working conditions and LTSA-MD. Physical working conditions refer to awkward body postures, such as repetitive movements and rotations, heavy physical exertion and lifting heavy loads.^34^ A Swedish study examined physical working conditions and found only working in a twisted position being associated with LTSA-MD in health care workers.^31^ Although work has generally evolved into more mentally and less physically demanding,^35^ physical working conditions are still prevalent in the lowest socioeconomic groups as well as in certain sectors, such as in health and social care work.

Working conditions are important in the prevention of LTSA-MD, as they are modifiable.^34,36,37^ However, the extent to which physical and psychosocial working conditions are related to younger employees’ LTSA-MD has so far been poorly understood. There are indications that younger employees may be even more prone to adverse psychosocial working conditions compared to their older counterparts.^38^ Young employees have been also shown to report more lower job control than older employees.^39^ A Finnish follow-up study that found no associations between low job control or high job demands and depression-related work disability in 18- to 35-year-old employees,^9^ however, demonstrated over a threefold risk of work disability due to mental disorders in the lowest educational group compared with the highest educational group, and this difference was the largest among the very youngest employees.

According to extensive research evidence, SA-MD are more common among women than men.^6,10,14,16,18,40^ A greater burden of mental disorders among women^1^ has been explained by the differences in health behavior and help seeking behaviour.^41,42^ This hypothesis has been supported by findings of mental health outcomes of different severity, namely sickness absence and suicide: while SA-MD are more prevalent among workers in female-dominated occupations, suicides are most common in men and in male-dominated occupations.^25^ Provided explanations for the gender differences additionally include the occupational characteristics, and the distribution of working conditions in the gendered labor market. Female-dominated occupations, such as occupations in education and health and social care, are more often mentally demanding^43^ and associated with mental health problems.^44^ In their jobs, women have been shown to be more exposed to high psychosocial job demands and low job control than men.^45^ Furthermore, gendered occupational status has been found to be a background factor explaining the distinctive mental health profiles of women and men.^25^ Increased risk for mental disorders in women has also been explained by the double burden faced by women, which is caused by the combination of domestic work and paid work.^46^ In addition to women being more exposed to different stressors, it has been suggested that also biological factors such as differences in a stress response between women and men, would partly explain the gender differences in common mental disorders.^47^

So far, research evidence on the risk groups and risk factors of younger emploees’ LTSA-MD is limited and incoherent. In the prevention of LTSA-MD, special attention should be paid to high-risk occupational groups. Current societal changes such as population aging cause a rapidly increasing need for workforce for the health and social care sector, in particular.^48^ To meet this growing need, improvements in the working conditions of this sector may be needed.^48^ In this study, we produce new information that can be applied in decision making and actions aimed at preventing LTSA-MD in younger employees. We employ a large occupational cohort data to investigate how psychosocial and physical working conditions are associated with LTSA-MD among younger public sector employees in different employment sectors.

## METHODS

### Participants

This study is part of the Young Helsinki Health Study^49^ which is an ongoing occupational cohort study of 18- to 39-year-old employees of the City of Helsinki. The City of Helsinki is the largest employer in Finland employing approximately 38,000 people annually, in different occupations and employment sectors.^50^ The study was launched in 2017 and is an extension of the Helsinki Health Study (HHS).^51^ The survey questionnaire includes wide-ranging information on health, its social determinants and well-being at work. Detailed information on the study is provided elsewhere.^49^

The target population of the study consisted of 11,459 employees who were born in 1978 or after, and who had a job contract of at least 50% of regular work hours per week and lasting at least four months before the start of the data collection. The data were collected in 2017 using online and mailed surveys. An additional shorter telephone interview was conducted to target those who were not reached within two months of first sending the survey and who had phone number available. In total, 5898 participants (response rate 51.5%) of the target population responded to the online survey (*n* = 3407), mailed surveys (*n* = 1704). An additional telephone interview (*n* = 787) with a shorter version of the survey was conducted among those who did not respond to the survey after reminders and had telephone number available. Analysis of non-response showed that men, manual workers, employees in the lowest income quartile, those who had part-time jobs, and those with more long-term sickness absence spells were more likely to respond somewhat less often. For example, the response rate was 59% for those belonging to the highest income quartile and 43% for those participants who belonged to the lowest income quartile.^49^ For those who reported having a full-time employment contract the response rate was 52% and for those with a part-time employment contract it was 48%. The observed differences between socioeconomic groups were thus relatively small and that the data represents the research population quite well.^49^ Of all participants 79% were women, which corresponds to the gender distribution of the target population: Around 80% of employees in the Finnish municipal sector are women.^52^ Similarly, the majority of the city's employees are women (about 80%) and 37% of them work in Health and Social Care Services.^53^

In the current study, we used information on female participants who had given their informed consent to link their survey responses to register data, and those who reported currently working full-time or part-time, or being on a family leave, unpaid maternity leave, study leave or mainly studying. We included only participants with complete data. Participants who declined the linkage (*n* = 747) or had a missing answer to the question (*n* = 35) were removed from the analytical sample. Also, those reporting being on a long sickness absence (over 6 months), disability pension, or on a rehabilitation subsidy (*n* = 26) at the time of the survey, or those who did not answer the question (*n* = 10) were excluded. As the additional telephone survey (*n* = 552) did not cover all the questions addressed in this study, these participants were additionally excluded from the study. Because one participant could have several missing values, the numbers overlap. Lastly, we excluded those participants who had incomplete data in any of the study variables (*n* = 320). Differences in the means of LTSA-MD between missing data (*n* = 772) and complete data (*n* = 3048) were tested and no statistical difference was found. The final analytic sample size was 3048.

### Ethical Approval

The Young Helsinki Health Study protocol was approved by the Ethics Committee of the Faculty of Medicine, University of Helsinki. The study was also granted permissions from the City of Helsinki authorities. Permission to link register data to the survey responses was granted by the register data holders. Linking of participants’ survey responses to register data is based on their informed consent.

## MEASUREMENT

### Exposures

#### Psychosocial Working Conditions

Psychosocial working conditions were derived from Karasek's Job Content Questionnaire (JCQ).^54^ We used the Framingham version of JCQ^54^ and took into consideration the weightings of the questions. Job demand was measured by five items (α = 0.74) and job control by nine items (α = 0.81). In the questionnaire, participants were asked to rate their psychosocial working conditions on the Likert scale ranging from 1 (strongly agree) to 5 (strongly disagree) for example, “How well the following statements describe your work? Do you agree or disagree with the statement?.” Instead of summary scales, weighted mean scores were calculated for the demand and control scales. We included only participants who had responded to at least half of the items, that is, two of the demand items and four of the control items and calculated their mean values based on the available data. To identify participants with poor psychosocial working conditions, the job demand scores were dichotomized by the highest quartile as in similar studies.^31,55^ In a similar way, the job control scores were dichotomized by the lowest quartile.

#### Physical Working Conditions

We examined physical working conditions with an 18-item inventory developed by the Finnish Institute of Occupational Health.^56^ The questions concerned i.a. awkward working positions, sitting, lifting, noise, dust, and dirtiness, and humidity and wetness. In the questionnaire participants were asked to estimate the extent to which different working conditions occur and bother them using a Likert scale (does not occur, occurs but does not bother at all, occurs and bothers to some extent, occurs, and bothers a lot). Three factors were obtained by factor analysis: computer work (α = 0.82), hazardous work exposures (α = 0.76), and physical workload (α = 0.83). We concentrated only on computer work and physical workload, as hazardous exposures were rare in our target group of public sector employees of which the majority worked in the health and social care and education sectors. Computer work factor consisted of working with a computer display terminal, using a mouse and sitting. Physical workload factor included heavy physical work load and lifting, repetitive movements and rotations, and awkward work postures. Similar to other studies,^31^ we dichotomized the factor scores by the highest quartile to identify the highest intensity of exposure.

### Outcome

LTSA-MD was measured by sickness allowance. In Finland, all permanent residents aged 16–67 years, regardless of their employment status, are entitled to sickness allowance, a compensation for loss of income due to incapacity for work. Shorter sickness absence that lasts less than 12 calendar days is covered by the employer. Longer sickness absence, lasting a maximum of 300 days, is covered by the Social Insurance Institution of Finland (SII), and requires a medical certificate. Thus, diagnosis-specific data are only available for the long-term sickness absence lasting more than 11 calendar days.

Information on LTSA-MD was derived from the register of the SII. The data were collected prospectively over one year from the time the participant had responded to the survey. The data included all the diagnoses that belonged to the class of mental and behavioral disorders (F00–F99) in the International Classification of Diseases (ICD-10). Register data were prospectively linked to the survey responses using national unique ID numbers which are assigned to every Finnish citizen at birth and to migrants when they get a residence permit.^47^ We counted the number of LTSA-MD days over 1-year (12 months) follow-up from the next day of returning the survey questionnaire.

### Employment Sector

Information on participants’ employment sector was obtained from the personnel register of the City of Helsinki. Participants were divided into three groups: health and social care (*n* = 1564, 51%), education (*n* = 1129, 37%), and other (*n* = 367, 12%).^49^ In the health and social care sector the most common occupations were: clinical nurse, community health nurse, and care worker. The most common occupations in the education sector were: day care worker, kindergarten teacher, and elementary school teacher. The “other sectors” group was very heterogeneous but the most common occupations were: youth worker, tram driver, and project manager. According to the nonresponse analysis study,^49^ the proportions of different sectors correspond to the distribution of the target population, and the employment sector did not affect survey response.^41^

### Covariates

We controlled for several factors potentially contributing to the association between working conditions and LTSA-MD. All information was self-reported^49^ and variables were treated as dichotomous, except for age and education. Of sociodemographic factors we controlled for gender (man / woman), marital status (cohabiting or married/other), and age (19 to 29, 30 to 34, 35 to 39). As only very few participants were younger than 25 years of age, the youngest age group comprised all participants younger than 30 years of age. In the regression analysis age was used as a continuous variable. Education was used as a three-categorical variable: low (comprehensive school, upper secondary school and vocational school), intermediate (a bachelor's degree) and high (a master's or doctoral degree). Lifestyle-related factors were also considered according to previous studies.^34,37,57^ Smoking was measured as current daily or casual smoking (yes/no), and binge drinking as consuming usually 6 or more units of alcohol on a single occasion once in a month or more often for women and once in a week or more often for men (yes/no).^58^ Low physical activity was measured by total metabolic equivalent (MET) hours for leisure and commuting in a week (≤28 />28). The lowest recommended limit value for low physical activity is 14 MET hours.^59^ As only 10% (*n* = 304) of all participants reported 14 MET hours or less, we devided the MET hours into quartiles^60^ and set the cut-off at the lowest quartile point (28 MET hours) to identify the lowest activity group. Obesity (body mass index [BMI] >30 /≤30) was calculated from the self-reported weight and height (kg/m^2^).

### Statistical Analyses

First, the number of sickness absence days per 100 person-years was calculated for descriptive purposes and was examined by covariates. Second, estimated marginal means and 95% confidence intervals were calculated for LTSA-MD days by the exposure groups of working conditions and the baseline characteristics by using generalized linear models. The differences were evaluated through the test of homogeneity of variances. Second, we used negative binomial distribution in the regression models to estimate the associations between working conditions and sickness absence days (count data) over a 1-year follow-up. Previous studies examining sickness absence as count data have used the Poisson distributions^61,62^ or negative binomial regression models.^9,63,64^ Because of a strong overdispersion in our outcome variable, the assumptions of the Poisson distribution were violated and therefore negative binomial regression was selected.

The association between each working condition and LTSA-MD was first adjusted for age and gender (base model). The second model added marital status and educational level to the first model. In the third model the associations were adjusted additionally for smoking, binge drinking, low physical activity and obesity. Participants in the nonexposure groups were treated as the reference category. The results are presented as rate ratios (RR) and 95% confidence intervals (95% CI). All analyses were carried out by using the SPSS statistical software (IBM SPSS Statistics 26.0.0.1).

### Test for Interactions and Sensitivity Analysis

Interaction test was conducted to examine whether the relationship between working conditions and LTSA-MD is modified by employment sector. Interactions were tested with main effects by adding each interaction term to the base model with adjustments for age: “physical workload × sector,” “computer work × employment sector,” “job demands × sector,” and “job control × employment sector.” All of the studied interaction terms were significant (*P* < 0.05). Based on these findings the results were stratified by the employment sector.

Previous mental health problems and past working conditions may affect LTSA-MD and cause overestimation bias. We conducted a sensitivity analysis to examine the possible overestimation problem by running an additional model in the negative binomial regression analysis. The association between working conditions and LTSA-MD was adjusted for physician diagnosed mental disorder that was measured in the survey with a question: “Have you ever had any of the following conditions diagnosed by your doctor: eating disorder, depression, anxiety, or other mental disorder?” (yes/no). The results of the sensitivity analysis are provided in the Supplemental Digital Content (supplemental Table S1).

## RESULTS

### Descriptive Results

The characteristics of the participants (*n* = 3048) are displayed in Table 1. Mean age was 32 years (range 19 to 39, standard deviation [SD] 4.6). Two thirds of the participants (66%) reported being married or cohabiting. Educational level was distributed in the followay: 30% had a vocational or upper secondary education degree, 40% a lower university degree and 30% had at least a master's degree. Almost a quarter of the participants reported current smoking (24%) and for 19% alcohol use habits that met the criteria for binge drinking. About 14% of the participants met the criteria of obesity (BMI ≥ 30). The mean body mass index was 25 (SD 5.4) and the median 24. The mean physical activity level among all participants was 60 MET hours (SD 51.4) and the median was 48.

**TABLE 1:** Baseline Characteristics of the Participants (*n* = 3048) and Long-Term Sickness Absence Days Due to Mental Disorders (> 11 Calendar Days) Over a 1-Year Follow-Up, Calculated Per 100 Person-Years

Variables	N (%)	Mean^∗^ (CI 95%)
Age		
19–29	1009 (33)	320 (298–343)
30–34	1049 (34)	201 (187–217)
35–39	990 (33)	154 (142–166)
All	3048 (100)	225 (216–235)
Educational level		
Low	927 (30)	290 (269–312)
Intermediate	1207 (40)	247 (231–264)
High	914 (30)	129 (119–141)
Cohabiting or married		
No	1027 (34)	353 (329–378)
Yes	2021 (66)	160 (151–169)
Low physical activity^†^		
No	2244 (74)	238 (226–250)
Yes	804 (26)	190 (174–207)
Current smoking		
No	2324 (76)	188 (179–198)
Yes	724 (24)	343 (316–373)
Binge drinking		
No	2457 (81)	202 (193–212)
Yes	591 (19)	319 (291–350)
Obesity		
No	2607 (86)	211 (201–221)
Yes	441 (14)	309 (277–344)
High physical workload^‡^		
No	2288 (75)	239 (228–251)
Yes	760 (25)	183 (167–200)
High exposure to computer work^‡^		
No	2287 (75)	194 (184–204)
Yes	761 (25)	318 (293–345)
High job demands^‡^		
No	2178 (71)	179 (169–188)
Yes	870 (29)	341 (316–368)
Low job control^†^		
No	2228 (73)	178 (169–187)
Yes	820 (27)	353 (327–382)
Employment sector		
Health and social care	1557 (51)	303 (286–321)
Education	1126 (37)	126 (117–137)
Other	365 (12)	196 (173–222)

95% CI, 95% Confidence intervals.

∗ Unadjusted mean.

† Lowest quartile.

‡ Highest quartile.

### Long-Term Sickness Absence Due to Mental Disorders

Table 1 further shows the mean duration of LTSA-MD per 100 person years by background characteristics. The prevalence of having at least one LTSA-MD during the 1-year follow-up was 4% (*n* = 116) in the total analytic sample. Among them, the mean length of the sickness absence was 59 days (SD 63.5, 95% CI 47.4–70.8), ranging from 1 to 312 days.

Mean duration of LTSA-MD days was higher in the youngest age group compared to the oldest age group, and among those reporting lowest educational attainment compared to the highest educational attainment. Additionally, participants who reported not being married or cohabiting had higher mean of LTSA-MD compared to those who reported being married. Mean LTSA-MD differed also by lifestyle factors. Those who reported current smoking, binge drinking, or BMI that met the criteria of obesity had higher mean on LTSA-MD. Of the work-related exposures, those who reported high job demands or and low job control had a higher mean duration of LTSA-MD compared to the reference groups. Clear differences were also found by the employment sector. The mean duration of LTSA-MD was over two-fold higher in the health and social care sector compared to the education sector and about one and a half times higher compared to the other sectors.

### Associations Between Adverse Working Conditions and Long-Term Sickness Absence Due to Mental Disorders

Tables 2–4 present rate ratios and confidence intervals (RR, 95% CI) for working conditions and LTSA-MD stratified by employment sector.

**TABLE 2:** Rate Ratios and Confidence Intervals for Adverse Working Conditions and Long-Term Sickness Absence Due to Mental Disorders (>11 Calendar Days) in the Health and Social Care Sector (*n* = 1557)

Health and Social Care Sector				
		Model 1. (RR, 95% CI)	Model 2. (RR, 95% CI)	Model 3. (RR, 95% CI)
Physical workload	High	0.64 (0.56–0.74)	0.48 (0.41–0.56)	0.47 (0.40–0.55)
Computer work	High	1.51 (1.34–1.70)	2.29 (2.00–2.62)	2.72 (2.35–3.14)
Job demands	High	2.56 (2.26–2.90)	2.49 (2.19–2.82)	2.41 (2.11–2.74)
Job control	Low	1.79 (1.58–2.02)	1.58 (1.38–1.82)	1.55 (1.34–1.79)

Dependent variable: sickness absence due to mental disorders (days/1-year follow-up). Fixed at the displayed value. Model 1. age. Model 2. age + marital status + education. Model 3. age + marital status + education + binge drinking + current smoking + low physical activity + obesity. 95% CI, confidence intervals; RR, rate ratio.

**TABLE 3:** Rate Ratios and Confidence Intervals for Adverse Working Conditions and Long-Term Sickness Absence Due to Mental Disorders (>11 Calendar Days) in the Education Sector (*n* = 1126)

Education Sector				
		Model 1. (RR, 95% CI)	Model 2. (RR, 95% CI)	Model 3. (RR, 95% CI)
Physical workload	High	1.11 (0.93–1.33)	0.79 (0.65–0.98)	0.68 (0.55–0.85)
Computer work	High	1.34 (1.05–1.73)	1.85 (1.41–2.43)	1.79 (1.36–2.36)
Job demands	High	1.01 (0.85–1.20)	1.11 (0.92–1.33)	1.15 (0.95–1.39)
Job control	Low	2.66 (2.20–3.20)	1.66 (1.36–2.04)	1.39 (1.11–1.73)

Dependent variable: sickness absence due to mental disorders (days/1-year follow-up). Fixed at the displayed value. Model 1. age. Model 2. age + marital status + education. Model 3. age + marital status + education + binge drinking + current smoking + low physical activity + obesity. 95% CI, confidence intervals; RR, rate ratio.

**TABLE 4:** Rate Ratios and Confidence Intervals for Adverse Working Conditions and Long-Term Sickness Absence Due to Mental Disorders (>11 Calendar Days) in the “other sectors” (*n* = 365)

Other Sectors				
		Model 1. (RR, 95% CI)	Model 2. (RR, 95% CI)	Model 3. (RR, 95% CI)
Physical workload	High	0.60 (0.40–0.90)	0.29 (0.18–0.46)	0.37 (0.23–0.61)
Computer work	High	1.18 (0.89–1.57)	3.00 (2.04–4.42)	3.48 (2.33–5.17)
Job demands	High	0.89 (0.63–1.26)	0.94 (0.64–1.38)	1.13 (0.75–1.68)
Job control	Low	0.77 (0.57–1.02)	0.62 (0.44–0.7)	0.60 (0.41–0.86)

Dependent variable: sickness absence due to mental disorders (days/1-year follow-up). Fixed at the displayed value. Model 1. age. Model 2. age + marital status + education. Model 3. age + marital status + education + binge drinking + current smoking + low physical activity + obesity. 95% CI, confidence intervals; RR, rate ratio.

#### Health and Social Care Sector

The associations between adverse working conditions and LTSA-MD among health and social care workers are shown in Table 2. Among health and social care workers, both physical and psychosocial working conditions were associated with LTSA-MD. However, high physical workload was negatively associated with LTSA-MD (RR 0.47, 0.40 to 0.55) compared to the reference group, when adjusting for age, education, marital status, body mass index, physical activity, smoking and binge drinking (model 3.). High exposure to computer work was strongly associated with higher LTSA-MD (RR 2.72, 2.35 to 3.14) in the fully adjusted model. Of psychosocial working conditions, high job demands were associated with LTSA-MD (RR 2.41, 2.11 to 2.74) and low job control with 1.55 (1.34 to 1.79) times greater RR compared to the reference group (model 3).

#### Education Sector

The associations of the working conditions and LTSA-MD in the education sector are presented in Table 3. Among education sector employees, high physical workload was negatively associated with LTSA-MD (RR 0.68, 0.55 to 0.85) compared to the reference group (model 3). High exposure to computer work was positively associated with LTSA (RR 1.79, 1.36 to 2.36) when compared to the reference group in the fully adjusted model. Of psychosocial working conditions only job control was associated with LTSA-MD. When controlling for all the confounding factors, low job control was associated with 1.92 (1.56 to 2.36) times higher RR compared to the reference group (model 3).

#### Other Sectors

Table 4 shows the results for the “other sectors.” In this group, high physical workload was negatively associated with LTSA-MD (RR 0.37, 0.23 to 0.61) when compared to the reference group (model 3). When all the covariates were taken into account, the role of computer work was highlighted and positively associated with LTSA-MD, with 3.48 (2.33 to 5.17) times higher RR compared to the low exposure group. Results of psychosocial working conditions were mixed. High job demands were not associated with LTSA-MD among workers in the other sectors. Low job control and LTSA-MD were negatively associated (RR 0.60, 0.41 to 0.86) when compared to the reference group with high control (model 3).

### Sensitivity Analysis

In the analytic sample (*n* = 3048) 24% of the participants reported having been diagnosed with a mental disorder at some point in their life. The results are described in the Supplemental Digital Content (S5). In the health and social care sector, the RR of working conditions and LTSA-MD mainly increased after controlling for mental disorder. Only for high job demands did the association slightly decrease. In the education sector, neither of the physical working conditions was associated with LTSA-MD when adjusting for prior mental disorder. The adjustment increased the associations of both high job demands and low job control and LTSA-MD. In the other sectors group, adjustment for prior mental disorder increased the RR of high exposure to computer work and strengthened the negative associations of both high physical workload and low job control and LTSA-MD. In this group, the adjustment for a prior mental disorder did not affect the relationship between high job demands and LTSA-MD as there was no association found.

## DISCUSSION

The present study examined the associations between working conditions and long-term sickness absence due to mental disorders (LTSA-MD). We used a large occupational cohort of 3048 younger (aged 19 to 39) female municipal sector employees from Finland, with a prospective linkage to the Social Insurance Institution of Finland's national records on medically certified LTSA-MD. To the best of our knowledge, this is the first study to examine the associations of both physical and psychosocial working conditions and mental disorders in younger employees by using record data of LTSA-MD.

Our main findings are as follows: First, we found associations between both psychosocial and physical working conditions and LTSA-MD. These associations were independent of lifestyle-related and socio-demographic factors that we controlled as covariates. Second, our study indicated differences between employment sectors in the exposure to adverse working conditions related to higher risk of LTSA-MD.

Our findings on the associations between physical working conditions and LTSA-MD were unexpected. Physical workload, referring to that is, repetitive movements and rotations, heavy lifting, and awkward work postures, was found negatively associated with LTSA-MD across all the employment sectors addressed in the study, meaning that experiencing high physical workload showed a protective effect against LTSA-MD among younger female employees. In contrast to this, high exposure to computer work, that is, sitting and using computer mouse and screen, was associated with increased LTSA-MD in all the sectors and proved to be an important factor in LTSA-MD among younger female employees. As far as we are aware of, there are no previous studies looking at the the association between computer work and LTSA-MD, or between adverse physical working conditions and LTSA-MD specifically among younger employees. However, our results on physical workload are inconsistent with previous studies, which have shown positive associations between twisted position and LTSA-MD among working-age health care workers,^31^ between physical workload and all-cause sickness absence among working population,^65–67^ and between physical working conditions and other mental disorder-related outcomes among midlife and older employees.^34,37^ Instead, our findings about computer work provide paraller indications to previous research regarding midlife and older municipal employees, in which computer work was found positively associated with disability retirement due to mental disorders.^68^ Computer-related work is constantly increasing in all occupations.^69,70^

Psychosocial working conditions were associated with LTSA-MD, however, these associations varied by employmet sector. High job demands were positively associated with LTSA-MD only in the health and social care sector. Low job control was positively associated with LTSA-MD in the health and social care sector and in the education sector. However, in the “other sectors” group, low job control was found negatively associated with LTSA-MD. This unexpected result may be explained by the small sample size and by the great heterogeneity of occupations in the group. The group included a wide range of different manual and non-manual occupations from food service and maintenance service workers to project managers and coordinators. occupation is shown to remarkably modify both physical and psychosocial working conditions.^71^ There is a lack of comparable studies on the subject and previous studies, however, our results on the health and social care sector and the education sector broadly support the evidence obtained from studies investigating working populations including all ages, as well as studies focusing on older employees.^29^

Previous studies in concentration on either employees of all ages, or on older employees, have demonstrated almost a twofold higher risk of LTSA-MD for employees in the health and social care sector compared to “other sectors”^10,28,72^ but also compared to other human service occupations.^72^ These results have also been found by other clinical mental disorder outcomes.^26,73,74^ The results of the current study support these findings, as we observed the highest rate of LTSA-MD in the health and social care sector. Our study confirmes that these employment sector-related differences exist also in the youngest employee groups. Interestingly, in our study health and social care sector had higher rate of LTSA-MD even compared to education sector, which is often considered to belong to the high risk occupational group of LTSA-MD, human service work.^72,75^ Similar findings have been shown in another Finnish longitudinal study, that showed higher relative risk of LTSA-MD for social workers compared to special education teachers or preschool teachers.^76^ The higher risk in the health and social care sector may be explained by the specific work characteristics of the sector, that constitute a mental disorder risk; such as traumatizing events and extreme emotional demands.^77–79^ Health and social care workers are also exposed to long working hours and shift work or night work, which have been associated with sleep disturbances.^80,81^ The need for preventive measures of LTSA-MD is emphasized in health and social care, where the employee's mental disorders are reflected in the safety and quality of patient work.^82–84^

### Strengths and Limitations

In this study, we used a large representative sample of 19 to 39-year-old Finnish female public sector employees. A key strength of our study was the ability to use national record data on medically certified sickness absence and prospectively link these data to survey responses. There was no loss to the follow-up in the record data and, record data is perceived as a reliable data source free from response and recall biases.^85^ In previous studies investigating the associations between working conditions and mental disorders these factors have mostly been self-reported. We measured mental disorders by medically certified LTSA-MD, which is considered to represent severe mental disorders.^86^ We were also able to use validated measures to assess physical and psychosocial working conditions.

The study has some limitations that should be considered when interpreting the results. First, record data on sickness absence do not directly reflect the state of health of the population as there are many factors contributing to sickness absence; factors related to legislation, social security and benefit system, economic cycles and the labor market.^87^ Practices for granting medically certified sickness absence are often unique for a country, and that limits the generalizability of our results.^22^ Mental disorders can also, due to their multifaceted nature and comorbidity, be hidden behind other illnesses or self-certified short sickness absence. These have been shown to be prevalent, especially in younger employees.^12^ Comorbidity is common among, and employees with mental disorders often have somatic symptoms.^88,89^ It has been shown that physicians may prefer granting sickness absence primarily for musculoskeletal diagnoses rather than for mental disorders.^31^ In addition, not all employees with even more severe mental disorders end up on sick leave, and many people who have mental disorders lack treatment.^90^

Second, we did not control whether a participant had had LTSA-MD prior to responding to the survey, and we were not able to select a cohort of employees whose sickness absence would start from the date of the survey. The long-term sickness absence due to mental disorders is often preceded by earlier symptoms of mental disorders and shorter sickness absence spells^91^ and to qualify for granted long-term sickness absence (ie, sickness allowance) due to mental disorders in Finland one must have a physician certified mental disorder-based diagnosis. The incidence of mental disorders is the highest in young adulthood and the median age of onset for depressive disorder is 24.^92^ The majority of the participants in our study were over 30 years of age and the average age was 32. Thus, it is likely that in most of the cases mental disorders have started considerably earlier than at our point of measurement. Furthermore, neither do the experiences of working conditions begin at the moment of our measurement point (baseline). Our participants may already have several years, the oldest even two decades, of work history as well as such life history that may affect the LTSA-MD or the experiences of current working conditions. However, this study does not seek to investigate causation or the incidence of sickness absence and our results on the statistical associations should not be interpreted as causal. Rather, we aim to explore the relationships between working conditions and sickness absence over the 1-year follow-up. We ran an additional sensitivity analysis to control the possible effects of prior mental disorders by controlling the associations for self-reported lifetime physician diagnosed mental disorder. As a result, the associations between working conditions and LTSA-MD mainly strengthened by the adjustment and remained broadly similar, which supports our findings. Nevertheless, the analysis showed diverse effects between sectors and would require a further examination.

Third, the healthy worker effect and selection into the labor market must be considered, especially in relation to younger workers and mental disorders, as mental disorders typically emerge in late adolescence or early adulthood.^93^

Fourth, working conditions were self-reported. Self-reported data are susceptible to response biases. In particular, mental distress can negatively impact on employees’ perceptions of working conditions.^35,94,95^ External measurement of psychosocial working conditions has been proved to be more strongly associated with all-cause sickness absence compared with self-resported measures in a large population-based study.^96^ This may have led to an underestimation of the results.

Fifth, another possible cause of underestimation could be the relatively short 1-year follow-up time, as we used survey data of the first wave of the Young Helsinki Health Study. This may have led to an underestimation of the effets of working conditions on LTSA-MD.

Sixth, in this study, the associations were adjusted for sociodemographic, socioeconomic and lifestyle factors, but not for health condition factors due to possible overlap between mental disorders and health: For example, sleep disturbances can be both predictors^97^ and symptoms^98^ of mental disorders. As in previous studies,^34^ we examined working conditions in separate statistical models, and hence it is not possible to compare or draw conclusions of their independent effects.^99^ It is also noteworthy that adjusting psychosocial working conditions for each other has been shown to attenuate their effects on self-reported diagnosed mental disorders.^100^

Seventh, the response rate was relatively low, 51.5%. However, this is in line with recent survey studies reporting low response rates.^101–104^ Declining response rates constitute an increasing challenge for epidemiological research.^49^ The response rates are shown to vary by individual factors, such as attitudes,^102^ age and sex,^105–115^ but also by countries.^104^ Low response rates contribute to possible nonresponse biases and the representativeness and generalizability of survey data.^102^ A nonresponse analysis^49^ of the present cohort indicated differences in the survey response between socioeconomic groups: the employees with a higher socioeconomic position and less long-term sickness absence were more likely to respond. Nevertheless, the observed differences were relatively small, and the participants represented the target group reasonably well.^49^ Similarly, a large body of evidence shows that the underrepresentation of lower socioeconomic classes in epidemiological studies is a common challenge.^105–122^

Finally, future research is needed to examine whether these results are generalizable to private sector employees, as well as to male-dominated employment sectors. In addition, our results of the other employment sectors may not be representative of other municipal sectors, as the group covered only a one-tenth of all participants and consisted of a wide range of different occupations.

## CONCLUSIONS

Based on our results employers should pay attention to working conditions when aiming to prevent mental disorders in younger female employees, as both psychosocial and physical working conditions were associated with long-term sickness absence due to mental disorders (LTSA-MD). The results of the study can be used to improve working conditions when aiming to prevent LTSA-MD in younger female employees. These prevention measures should be targeted at high risk groups of LTSA-MD, such as the employees in the health and social care sector.

## Supplementary Material

Supplemental Digital Content
