# Supplementary material for: Working Conditions and Long-Term Sickness Absence Due to Mental Disorders: A Prospective Record Linkage Cohort Study Among 19- to 39-Year-Old Female Municipal Employees
Source: J Occup Environ Med. 2021 Oct 28;64(2):105–14. doi: 10.1097/JOM.0000000000002421 (PMC8812422; doi:10.1097/JOM.0000000000002421)
Supplement: Supplemental Digital Content [file joem-64-105-s001.docx]

| **S1.** Rate ratios and confidence intervals for adverse working conditions and long-term sickness absence due to mental disorders (> 11 calendar days) adjusted for self-reported physician diagnosed mental disorder | | | | |
| --- | --- | --- | --- | --- |
| **Fully adjusted associations by employment sector** | | | | |
|  | | **Health and social care** sector (n = 1,474)  RR (95% CI) | **Education**  (n = 1,057)  RR (95% Cl) | **Other sectors**  (n = 351)  RR (95% CI) |
| **Physical workload** | High | 0.55 (0.47–0.66) | 0.96 (0.76–1.20) | 0.20 (0.11–0.37) |
| **Computer work** | High | 3.70 (3.14–4.36) | 1.34 (0.98–1.84) | 6.54 (4.13–10.35) |
| **Job demands** | High | 2.36 (2.04–2.74) | 1.41 (1.15–1.72) | 1.45 (0.94–2.24) |
| **Job control** | Low | 2.51 (2.14–2.95) | 2.51 (2.14–2.95) | 0.32 (0.20–0.50) |

| Dependent Variable: sickness absence due to mental disorders (days/1-year follow-up). Fixed at the displayed value.  RR, Rate ratio  95% CI, Confidence intervals |
| --- |
| Model adjustments: age + marital status + education + mental disorder diagnosis + binge drinking + current smoking + low physical activity + obesity |
